# The GFAP proteoform puzzle: How to advance GFAP as a fluid biomarker in neurological diseases

**DOI:** 10.1111/jnc.16226

**Published:** 2024-09-17

**Authors:** Dea Gogishvili, Madison I. J. Honey, Inge M. W. Verberk, Lisa Vermunt, Elly M. Hol, Charlotte E. Teunissen, Sanne Abeln

**Affiliations:** ^1^ Bioinformatics, Computer Science Department Vrije Universiteit Amsterdam Amsterdam The Netherlands; ^2^ AI Technology for Life, Department of Computing and Information Sciences, Department of Biology Utrecht University Utrecht The Netherlands; ^3^ Neurochemistry Laboratory, Department of Laboratory Medicine Amsterdam University Medical Centers, Amsterdam Neuroscience, Vrije Universiteit Amsterdam The Netherlands; ^4^ Department of Translational Neuroscience, UMC Utrecht Brain Centre University Medical Centre Utrecht, University Utrecht Utrecht The Netherlands

**Keywords:** biology, biomarker, GFAP, immunoassay, proteoform, structure

## Abstract

Glial fibrillary acidic protein (GFAP) is a well‐established biomarker of reactive astrogliosis in the central nervous system because of its elevated levels following brain injury and various neurological disorders. The advent of ultra‐sensitive methods for measuring low‐abundant proteins has significantly enhanced our understanding of GFAP levels in the serum or plasma of patients with diverse neurological diseases. Clinical studies have demonstrated that GFAP holds promise both as a diagnostic and prognostic biomarker, including but not limited to individuals with Alzheimer's disease. GFAP exhibits diverse forms and structures, herein referred to as its proteoform complexity, encompassing conformational dynamics, isoforms and post‐translational modifications (PTMs). In this review, we explore how the proteoform complexity of GFAP influences its detection, which may affect the differential diagnostic performance of GFAP in different biological fluids and can provide valuable insights into underlying biological processes. Additionally, proteoforms are often disease‐specific, and our review provides suggestions and highlights areas to focus on for the development of new assays for measuring GFAP, including isoforms, PTMs, discharge mechanisms, breakdown products, higher‐order species and interacting partners. By addressing the knowledge gaps highlighted in this review, we aim to support the clinical translation and interpretation of GFAP in both CSF and blood and the development of reliable, reproducible and specific prognostic and diagnostic tests. To enhance disease pathology comprehension and optimise GFAP as a biomarker, a thorough understanding of detected proteoforms in biofluids is essential.

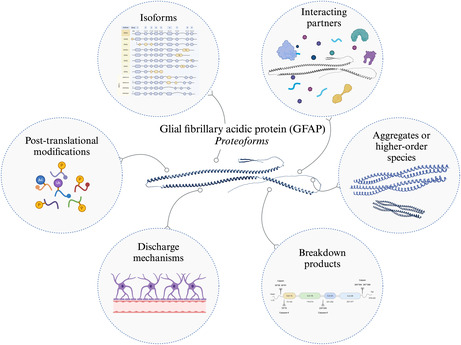

## Graphical abstract

Glial fibrillary acidic protein (GFAP) is a well‐established biomarker of reactive astrogliosis in the central nervous system. In this review, we explore how the physicochemical characteristics of GFAP may influence its detection, which may underlie the differential diagnostic performance of GFAP in different biological fluids and can provide valuable insights into underlying biological processes. We summarise how knowledge of GFAP biology and proteoforms, including isoforms, post‐translational modifications, discharge mechanisms, breakdown products, higher‐order species and interacting partners, can be utilised to guide future biomarker test development and enhance disease pathology comprehension.

**Abbreviations**
3D: 3‐dimensional
AA: amino acid
aCSF: artificial cerebrospinal fluid
AD: Alzheimer's disease
ALS: amyotrophic lateral sclerosis
APP: amyloid precursor protein
AUC‐ROC: area under the curve of the receiver operating characteristic curve
AxD: alexander disease
BBB: blood–brain barrier
BTI: brain trauma indicator
CNS: central nervous system
CSF: cerebrospinal fluid
DLB: dementia with lewy bodies
EV: extracellular vesicle
FT: freeze–thaw
FTD: frontotemporal dementia
FTLD: frontotemporal lobar degeneration
GFAP: glial fibrillary acidic protein
HD: Huntington's disease
HPA: human protein Atlas
IF: intermediate filament
KPBS: potassium phosphate buffer saline
MS: multiple sclerosis
MSD: Meso Scale Discovery
NfL: neurofilament light
PAD2: peptidylarginine deaminase 2
PD: Parkinson's disease
PET: positron emission tomography
PTM: post‐translational modification
sHSP: small heat shock protein
TBI: traumatic brain injury

## 1 INTRODUCTION

Glial fibrillary acidic protein (GFAP) is an essential component of the cytoplasmic intermediate filament cytoskeleton in astrocytes, facilitating structural integrity, motility, signal transduction and cell homeostasis (Abdelhak et al., 2018; Emirandetti et al., 2006; Kawajiri et al., 2003; Lowery et al., 2015; Messing et al., 1998; Rutka et al., 1994; Yoshida et al., 2007). Following injury, disease or infection of the central nervous system (CNS), levels of GFAP increase (Abdelhak et al., 2022; Heimfarth et al., 2022; Messing & Brenner, 2020; Mondello et al., 2021). In the context of brain injury and various CNS pathologies, astrocytes undergo significant morphological, molecular and functional changes and are termed as ‘reactive astrocytes’ (Escartin et al., 2021). GFAP is, therefore, a widely used biofluid‐ and tissue‐based biomarker of reactive astrogliosis in the CNS since its expression in the brain is astrocyte‐specific and strictly regulated after damage and during disease (Colangelo et al., 2014; Eddleston & Mucke, 1993; Middeldorp & Hol, 2011).

GFAP protein levels can be measured within a detectable range in human biofluids. The measurement of GFAP as a blood‐based biomarker was facilitated by the advent of ultra‐sensitive technologies to detect proteins at biologically relevant concentrations, resulting so far in a large number of studies examining levels of GFAP in clinical samples from patients with different neurological diseases (Abdelhak et al., 2022; Ishiki et al., 2016; Oeckl et al., 2022; Teunissen et al., 2022).

Given that cerebrospinal fluid (CSF) is in direct contact with the brain, it is considered to more accurately and acutely reflect neuropathological changes compared to blood (Aluise et al., 2008). Moreover, peripheral protein sources and transport of brain‐derived proteins across the blood–brain barrier may hamper biomarker detection and result in smaller measurable fold‐changes compared to those measured in CSF (Olsson et al., 2016; Schindler et al., 2019). Thus, brain‐specific proteins, such as GFAP, are generally expected to exhibit better performance as biomarkers in CSF compared to blood (Palmqvist et al., 2020; Simrén et al., 2022). However, for GFAP this is not so clear cut.

The diagnostic value of GFAP varies across biological fluids and neurological diseases. For instance, CSF GFAP shows superior diagnostic performance for Alexander disease (AxD) compared to plasma GFAP (Jany et al., 2015; Kyllerman et al., 2005; Schmidt et al., 2013). A particularly striking finding is that GFAP measured in plasma has a better discriminative performance to distinguish between individuals with and without amyloid pathology across the Alzheimer's disease (AD) clinical continuum compared to GFAP measured in CSF (Baiardi et al., 2022; Benedet et al., 2021; Simrén et al., 2022). Additionally, serum GFAP shows a stronger negative correlation with mini‐mental state examination scores compared to CSF GFAP in a cohort of patients with different types of dementia (Oeckl et al., 2019). These discrepancies between CSF and blood measurements, as well as the secretion mechanism of GFAP from astrocytes to these matrices, are not fully understood.

Despite the increasing scientific interest in GFAP, the implications of its proteoforms are largely unknown. The proteoform properties of GFAP, such as its 3‐dimensional (3D) structure, discharge mechanisms into different body fluids, breakdown products, intermediate filament network and post‐translational modifications (PTMs) may affect its ability to be detected in different matrices. The highly flexible nature of GFAP and the potential impact of its proteoforms on clinical assays emphasise the importance of targeted strategies.

In this article, we aim to provide a comprehensive overview of protein characteristics and highlight knowledge gaps and respective shortcomings of available biomarker tests for GFAP detection and quantification. Based on these, we suggest future directions to improve the understanding of GFAP as a biomarker for various brain disorders, which could improve its clinical utility. To set the stage we first summarise the biology and structural properties of GFAP, and address key findings concerning its diagnostic and prognostic value as a biomarker measured in CSF, serum and plasma. The main section of this review delves into the current understanding and complexity of the protein structure, its physio‐chemical characteristics, and solvent accessibility. We discuss the properties of assays used to detect GFAP, and how antibody attributes translate to measurable levels of GFAP in biofluids. We demonstrate how proteoforms have been successfully utilised for the detection of other neurological biomarkers. Additionally, we provide hypotheses on how these challenges can be overcome and subsequent recommendations for the future development of robust assays targeting specific proteoforms of GFAP.

## 2 GFAP BIOLOGY

**FIGURE 1:**
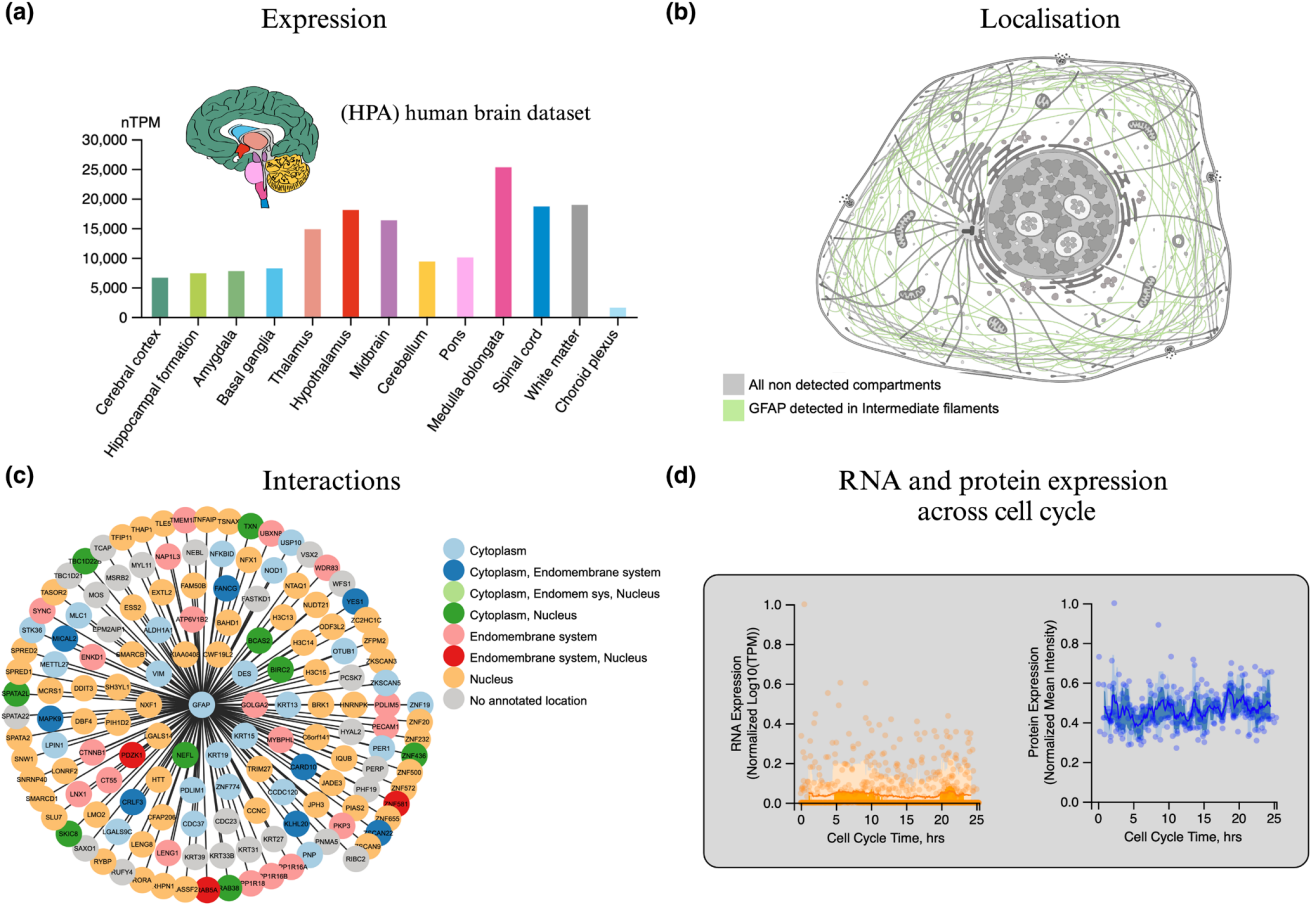
Biology of GFAP curated by Human Protein Atlas (HPA). GFAP is a highly dynamic structural protein involved in a plethora of biological processes, including but not limited to maintaining the integrity of the blood–brain barrier (BBB; Liedtke et al., 1996). (a) Brain‐specific expression of GFAP based on RNA consensus dataset consists of normalised expression levels of 13 brain regions (Uhlén et al., 2015). (b) GFAP localisation characterised by presence in all tested cells (Thul et al., 2017). (c) Interaction summary network of GFAP. The thickness of the edges represents the confidence of the interaction and nodes are coloured according to subcellular location. (d) GFAP RNA and protein expression are highly regulated during the cell cycle as GFAP is essential for the remodelling of glial frameworks in mitosis (Kawajiri et al., 2003; Messing et al., 1998; Rutka et al., 1994; Yoshida et al., 2007). The RNA expression level was determined by single‐cell RNA sequencing of the U‐2 OS FUCCI cell line. This cell line is a variant of the human cervical carcinoma cell line HeLa. Protein expression was determined by indirect immunofluorescence microscopy in the U‐2 OS FUCCI cell line. Normalised RNA and protein expression in individual cells is plotted along a linear representation of cell cycle pseudotime, as determined from the fluorescence intensities of the cell cycle markers (Karlsson et al., 2021).

**FIGURE 2:**
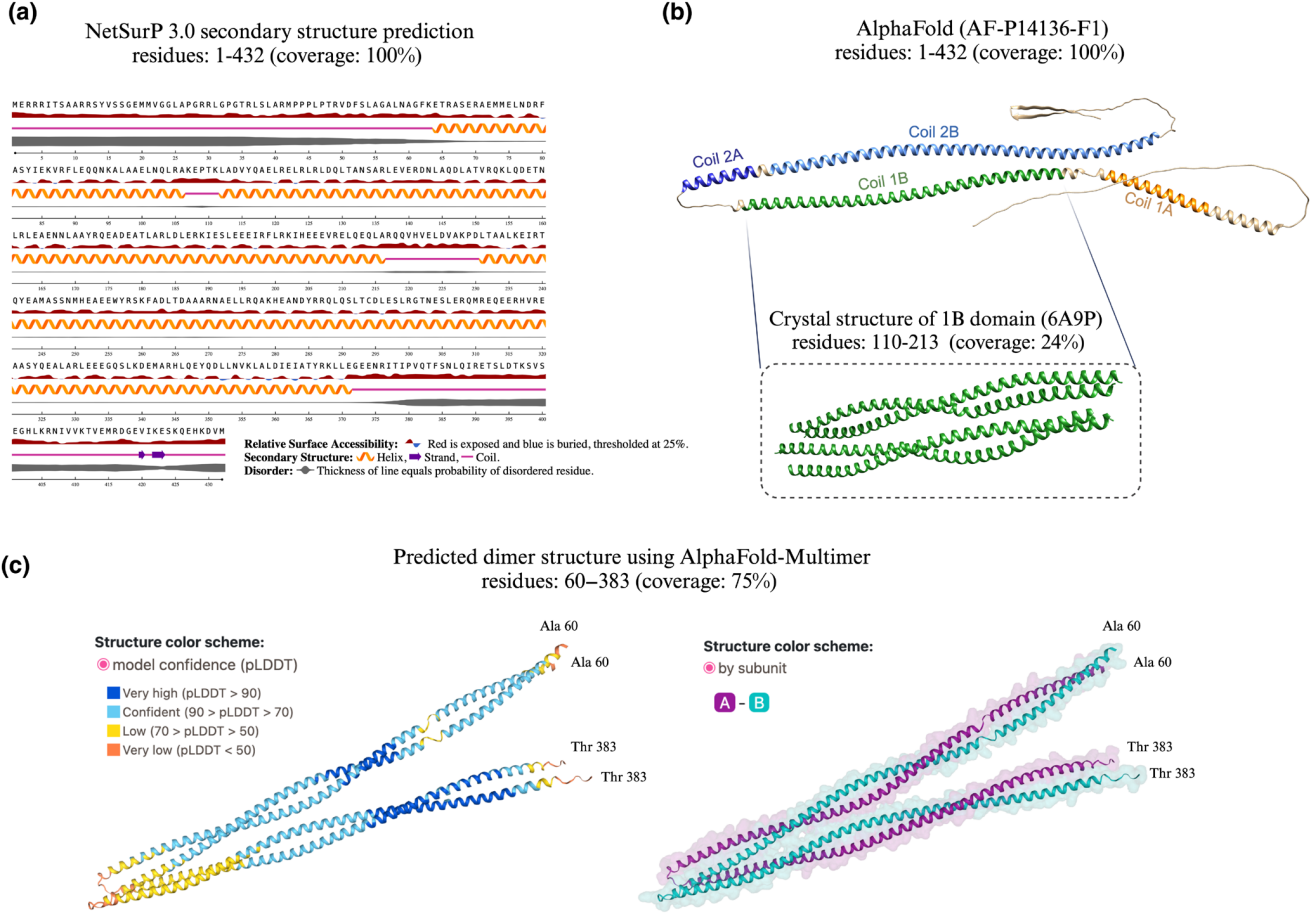
Structural characteristics of mono‐ and dimeric GFAP. Our understanding of the 3‐dimensional (3D) structure of GFAP is limited. (a) The predicted secondary structural components of GFAP using NetSurfP 3.0 covering 100% of the full‐length GFAP AA sequence (Høie et al., 2022; Klausen et al., 2019). (b) The 3D structure of GFAP predicted by AlphaFold covering 100% of the full‐length protein sequence composed of four coils: 1A, 1B, 2A, 2B (Jumper et al., 2021). Below the AlphaFold structure, the X‐ray PDB structure of the 1B domain of GFAP is displayed and determined to form a homotetramer covering 24% of the protein sequence (Kim et al., 2018). (c) The predicted dimer structure of recombinant GFAP using AlphaFold‐Multimer (Evans et al., 2021; Gogishvili et al., 2023) visualised with the PAE viewer tool (Elfmann & Stülke, 2023). Data obtained from hydrogen‐deuterium exchange measurements support the existence of this structure (Gogishvili et al., 2023).

GFAP is a signature intermediate filament (IF) type III protein for astrocytes (Yang & Wang, 2015), but is also expressed in peripheral glia (Kato et al., 1990), enteric glia (Grundmann et al., 2019) and Schwann cells (Hainfellner et al., 2001). The expression of GFAP is higher in white matter compared to grey matter astrocytes, therefore GFAP is highly expressed in regions rich in white matter, such as the medulla oblongata and the hypothalamus (Figure 1a). The amino acid (AA) sequence of GFAP is similar to other IF proteins with a shared central *α*‐helical (rod) domain flanked by the disordered amino‐ (head) and carboxy‐terminal (tail) domains that largely vary in AA sequence (Chernyatina et al., 2015). GFAP emerged early in the evolution of vertebrates and shows a high degree of conservation across species, with 90% identity between humans and mice and 67% identity between humans and zebrafish (Messing & Brenner, 2020; Nielsen & Jørgensen, 2003). Under physiological conditions, type III IFs assemble into large oligomers that can be visualised by electron microscopy (Parry & Steinert, 1999). A proposed multistep mechanism involves the parallel interactions of monomers through the coiled‐coil region, followed by an antiparallel association of dimers through the core rod domain (composed of four coils; 1A, 1B, 2A, and 2B), leading to the lateral association of tetramers to form octamers. These octamers then aggregate into mature filament structures containing 30–59 monomers in cross‐section (Messing & Brenner, 2020; Parry & Steinert, 1999). A type III IF protein—vimentin, which is a more flexible homologue of GFAP (Kim et al., 2018), serves as a prototypical model for the assembly of other proteins within this family. Similar to GFAP, vimentin expression is up‐regulated in reactive astrocytes (Ridet et al., 1997) and it has similarly been shown to form an antiparallel tetramer structure (Chernyatina et al., 2012). Crystal structures of the GFAP rod 1B domain have revealed a homotetramer architecture, composed of two parallel coiled coils stabilised by salt bridges and hydrophobic interactions (Figure 2a; Kim et al., 2018). However, the rod 1B domain represents only a fraction of the entire GFAP protein, and the native assembly of GFAP remains elusive (Figure 2b). Cryo‐electron tomography experiments have revealed the structure of polymerised vimentin filaments, which are comprised of five protofibrils each having 40 polypeptide chains in cross‐section (Eibauer et al., 2021). Although the 3D structure of GFAP is still not fully understood, a recent study has indicated that GFAP exists in various conformational species and that its dimer structure remains intact under strong denaturing conditions (Gogishvili et al., 2023; Figure 2c). This suggests that GFAP's structural flexibility under different conditions may play a role in its surface accessibility and ultimately function.

Like other type III IF proteins, GFAP is a highly dynamic structural protein involved in the formation of the cytoskeleton (Figure 1b). Having a large interactome (Figure 1c), GFAP is involved in various cellular processes, including (*i*) cell motility and migration (Yoshida et al., 2007), (*ii*) remodelling glial frameworks in mitosis, essential for cell proliferation, during which GFAP RNA and protein expression are highly regulated (Figure 1d; Kawajiri et al., 2003; Messing et al., 1998; Rutka et al., 1994; Yoshida et al., 2007), (*iii*) exocytosis and vesicle mobility (Potokar et al., 2007), (*iv*) synapse formation (Emirandetti et al., 2006), neuronal plasticity (Emirandetti et al., 2006), neurite outgrowth (Rozovsky et al., 2002) and neuronal sprouting (Finch, 2003), (*v*) the maintenance of CNS myelination (Giménez y Ribotta et al., 2000; Liedtke et al., 1996), and (*vi*) maintaining the integrity of the blood–brain barrier (BBB; Liedtke et al., 1996). Multiple studies have demonstrated that GFAP and vimentin knockout mice are more susceptible to severe long‐term consequences following brain injury, such as ischemic brain damage (Nawashiro et al., 1998, 2000), demonstrating the protective astrocytic function related to GFAP.

## 3 GFAP AS A BIOMARKER IN BRAIN DISORDERS

The activation of common inflammatory pathways is linked to early stages of neuropathological processes (Colangelo et al., 2014). Astrogliosis—glial activation, proliferation (present in acute damage), and increased GFAP expression were shown to be important to recover from initial damage during CNS injury. Nevertheless, such processes can become harmful in severe stress conditions (Kumar et al., 2021). Following acute damage such as spinal cord and traumatic brain injury (TBI), GFAP is up‐regulated immediately, and has been shown to reach a peak in the blood at 20 h following TBI (Papa et al., 2016, 2023), and a peak in both CSF and blood during the first 24–36 h after spinal cord injury (Kwon et al., 2010; Leister et al., 2023), subsequent to which levels decrease. In the context of chronic CNS injury, such as dementia, elevation of plasma GFAP levels begins 10–20 years prior to the onset of symptoms and neurodegeneration, and this rise in GFAP concentration continues throughout the dementia continuum (Chatterjee et al., 2023; Guo et al., 2024; Montoliu‐Gaya et al., 2023). Because of this early and sustained increase in GFAP levels, plasma GFAP has excellent prognostic value for conversion to dementia (Verberk et al., 2020). This difference in the time course of GFAP in acute compared to chronic events may be a reflection of the molecular and functional astrocytic changes in response to acute neuronal injury compared to chronic neurodegenerative disease pathology.

Different aspects of reactive astrogliosis and distinct subtypes of astrocytes may also underlie the difference in measurement of astrocytes in the living brain by positron emission tomography (PET) compared to using GFAP as a fluid biomarker. ^11^C‐DED is the gold‐standard PET radiotracer for imaging reactive astrogliosis and is a selective inhibitor of monoamine oxidase type B, expression of which increases in reactive astrocytes (Ekblom et al., 1993). A negative association was observed between plasma GFAP and ^11^C‐DED binding in autosomal dominant and sporadic AD brains (Chiotis et al., 2023). As such, it is proposed that ^11^C‐DED binding may reflect a ‘first‐wave’ of reactive astrogliosis, potentially in response to pre‐plaque soluble amyloid, whereas GFAP measured in biofluids may reflect more advanced amyloid pathology in AD progression, and thus a later reactive astrogliosis process (Fontana et al., 2023). Measurement of plasma GFAP and ^11^C‐DED binding in the context of other neurodegenerative diseases can help elucidate the relationship between these measures, functional astrocytic changes and other types of neuropathology.

**FIGURE 3:**
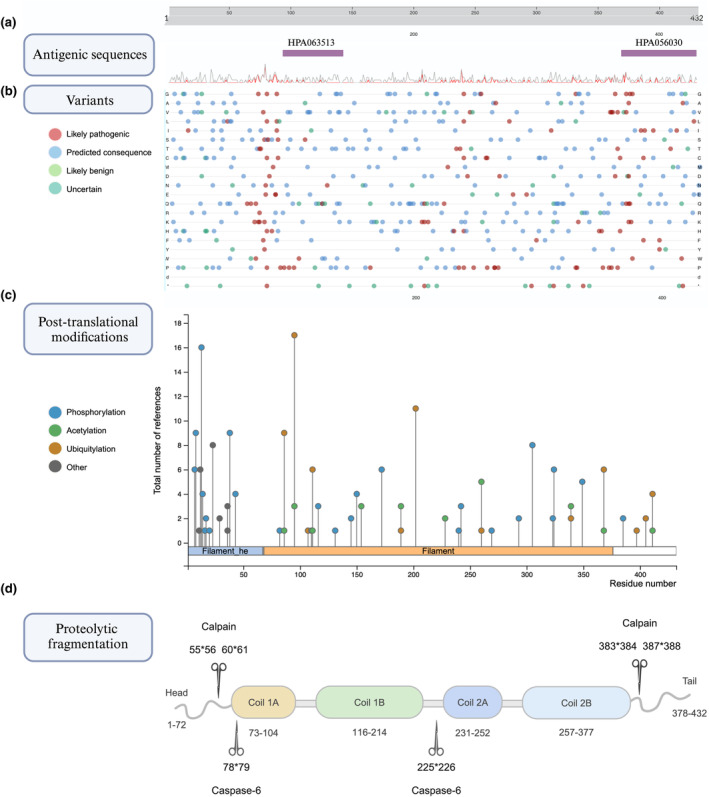
GFAP feature overview and breakdown products. GFAP and its modified or cleaved products play a key role in various cellular processes. GFAP epitopes which are targeted by commercially available immunoassays are mostly poorly characterised. (a) Demonstrates known antigenic sequences along the full‐length GFAP (antibodies targeting GFAP: HPA063513; HPA056030) and (b) mutations curated by UniProt (Consortium, T. U, 2023). Mutations and types of modifications are colour‐coded. (c) Protein post‐translational modifications (PTMs) functionally regulate the localisation, activity and assembly of GFAP. The visualisation of PTMs is based on PhosphoSitePlus (Hornbeck et al., 2014). (d) Full‐length GFAP is susceptible to proteolysis by calpain and caspase enzymes. A schematic representation of the proteolytic fragmentation of GFAP is shown. GFAP is shown as a linear model and major calpain and caspase 6 cleavage sites are indicated with scissors, whereby asterisks show between which amino acids the cleavage sites are. Adapted from Yang et al. (2022).

AxD is a rare disorder specific to astrocytes neuropathologically defined by Rosenthal fibres, which are aggregates of GFAP. This disease is caused by de novo mutations in the gene encoding GFAP, the majority of which are coding for regions located in the central rod domain (69–377 AA) of the protein (Messing,&amp;#x000A0;^2018^; Figure 3b). CSF GFAP levels are increased in AxD patients compared to controls (Jany et al., 2015; Kyllerman et al., 2005; Schmidt et al., 2013). The same effect has not been demonstrated for blood‐based GFAP, which showed no significant difference between controls, infantile‐, juvenile‐ and adult‐onset AxD patients, although this has only been investigated in one study to date (Jany et al., 2015). Reactive astrogliosis has been linked to many other CNS diseases, such as AD, Parkinson's disease (PD), frontotemporal dementia (FTD), amyotrophic lateral sclerosis (ALS), dementia with Lewy bodies (DLB), multiple sclerosis (MS), Huntington's disease (HD), and glioma (Glass et al., 2010; Jiwaji & Hardingham, 2022; van Asperen, Fedorushkova, et al., 2022). Elevated GFAP levels have, therefore, been found in the CSF of patients with various neurodegenerative diseases compared to controls (Axelsson et al., 2011; Oeckl et al., 2019). Blood‐based GFAP generally displays a similar pattern, with increases shown in AD, PDD, DLB and FTD cases compared to controls (Tang et al., 2023; Thijssen et al., 2022), and serum GFAP has been shown to distinguish between MS phenotypes (Ayrignac et al., 2020; Högel et al., 2020). In addition to being a promising diagnostic biomarker for various neurodegenerative diseases, GFAP can also be utilised for prognostic applications: rate of cognitive decline and higher risk of conversion to dementia (Benedet et al., 2021; Cicognola et al., 2021; Cullen et al., 2021; Verberk et al., 2020).

As evidenced, the measurements of GFAP in different biological fluids are not always equivalent across different CNS diseases. For instance, CSF GFAP is a better diagnostic biomarker in AxD whereas plasma GFAP has been demonstrated to outperform CSF GFAP for differentiation of amyloid‐positive and amyloid‐negative individuals in the context of AD. These distinctions emphasise the importance of context‐specific evaluation of GFAP levels, highlighting the need for tailored diagnostic strategies.

## 4 COMMERCIALLY AVAILABLE GFAP IMMUNOASSAYS

Before delving into GFAP's proteoform complexity, it should be disclosed that the epitopes which are targeted by commercially available immunoassays are mostly unknown or poorly characterised (Figure 3a; Waury et al., 2022). The assay with the most evidence concerning the antibodies used is the Quanterix Simoa singleplex or multiplex GFAP assay. This assay is widely used in clinical research and utilises antibodies from Banyan Biomarkers. The capture antibody is a mouse monoclonal IgG antibody (clone 2H12) and the detector antibody is a rabbit polyclonal antibody raised against the midsection of full‐length GFAP (Papa et al., 2012). Two epitopes for the capture antibody within human GFAP are reported, neither of which are entirely conserved between rat and human GFAP, or mouse and human GFAP (Zoltewicz et al., 2012). Both antibodies have been shown to recognise full‐length GFAP and a range of GFAP breakdown products varying in size from 48 to 38 kDa (Zoltewicz et al., 2012). Since both full‐length GFAP and various breakdown products are recognised with this assay (Zoltewicz et al., 2012), the antibodies likely bind to epitopes within the central rod domain (69–377 AA).

The Banyan Biomarkers' Brain Trauma Indicator (BTI) is an in‐vitro diagnostic test for the measurement of GFAP in the serum of suspected mild patients with traumatic brain injury. The BTI received a breakthrough device marketing authorisation from the FDA in 2018 (US Food and Drug Administration, 2018). In the decision memorandum from the FDA, it is demonstrated that the assay shows cross‐reactivity to NfL, but to no other proteins with similar homology to GFAP (US Food and Drug Administration, 2018). As such, a limitation of the procedure listed on the Banyan BTI Package Insert describes that because of the cross‐reactivity of neurofilament light (NfL) with the antibodies in the Banyan GFAP Kit, patients with neurodegenerative diseases such as Guillain‐Barré syndrome, ALS, PD, AD, or Creutzfeldt‐Jakob disease may have erroneously high Banyan GFAP, hence a false‐positive result.

Another GFAP biomarker test includes the NeuroToolKit from Roche, in which several biomarkers including GFAP can be measured in CSF or blood using a panel of automated exploratory prototype sandwich immunoassays (Johnson et al., 2023). Roche has developed a research‐use‐only GFAP electrochemiluminescence immunoassay to be used on the cobas e 801 and cobas e 402 immunoassay analysers. This assay uses monoclonal recombinant capture and detector antibodies; however, it is unknown which GFAP epitopes these antibodies target (Mayer et al., 2013). Other commercial GFAP immunoassays are the R‐Plex and S‐plex assays from Meso Scale Discovery (MSD; Kivisäkk et al., 2023; Spanos et al., 2022). These MSD assays utilise mouse monoclonal antibodies as both capture and detector antibodies, which were raised against the full‐length GFAP protein and show cross‐reactivity to mouse and rat GFAP protein (Kivisäkk et al., 2023; Spanos et al., 2022). No other information concerning these assays is publicly available.

To summarise, relatively little is known about which GFAP proteoforms are being targeted in commercially available immunoassays. The lack of detailed information about antibodies makes it challenging to compare studies and interpret discrepancies. This leaves plenty of room for improving our strategies to accurately measure GFAP to first unravel its function in brain pathologies and take advantage of this knowledge for developing specific biomarker tests.

## 5 PROTEOFORM COMPLEXITY

Given the major potential of GFAP as a biomarker of reactive astrogliosis for neurodegenerative and neurological diseases, as well as glioma, and its implementation in clinical settings (Abdelhak et al., 2022; Glass et al., 2010; Heimfarth et al., 2022; Jiwaji & Hardingham, 2022; Messing & Brenner, 2020; Mondello et al., 2021; van Asperen, Fedorushkova, et al., 2022), it is paramount to understand how the physio‐chemical characteristics of GFAP may influence its detection which may underlie the differential diagnostic performance of GFAP in different matrices. Although GFAP can be quantified well with commercially available immunoassays, it is unclear which GFAP proteoforms are being targeted with the available antibodies. Proteoforms of GFAP are altered in disease (Battaglia et al., 2019; Herskowitz et al., 2010; Ishigami et al., 2015; Kamphuis et al., 2014; Korolainen et al., 2005; Lin et al., 2021; Nicholas et al., 2004; Porchet et al., 2003) and could underly discrepancies between CSF and blood GFAP performance as a biomarker across various CNS diseases. Furthermore, targeting specific GFAP proteoforms could therefore result in disease‐specific biomarker tests. In the following section, we delve into the proteoform complexity of GFAP, which covers GFAP isoforms, PTMs, half‐life and breakdown products, surface accessibility, including structural flexibility and aggregation patterns, and protein–protein interactions.

### 5.1 Isoforms

**FIGURE 4:**
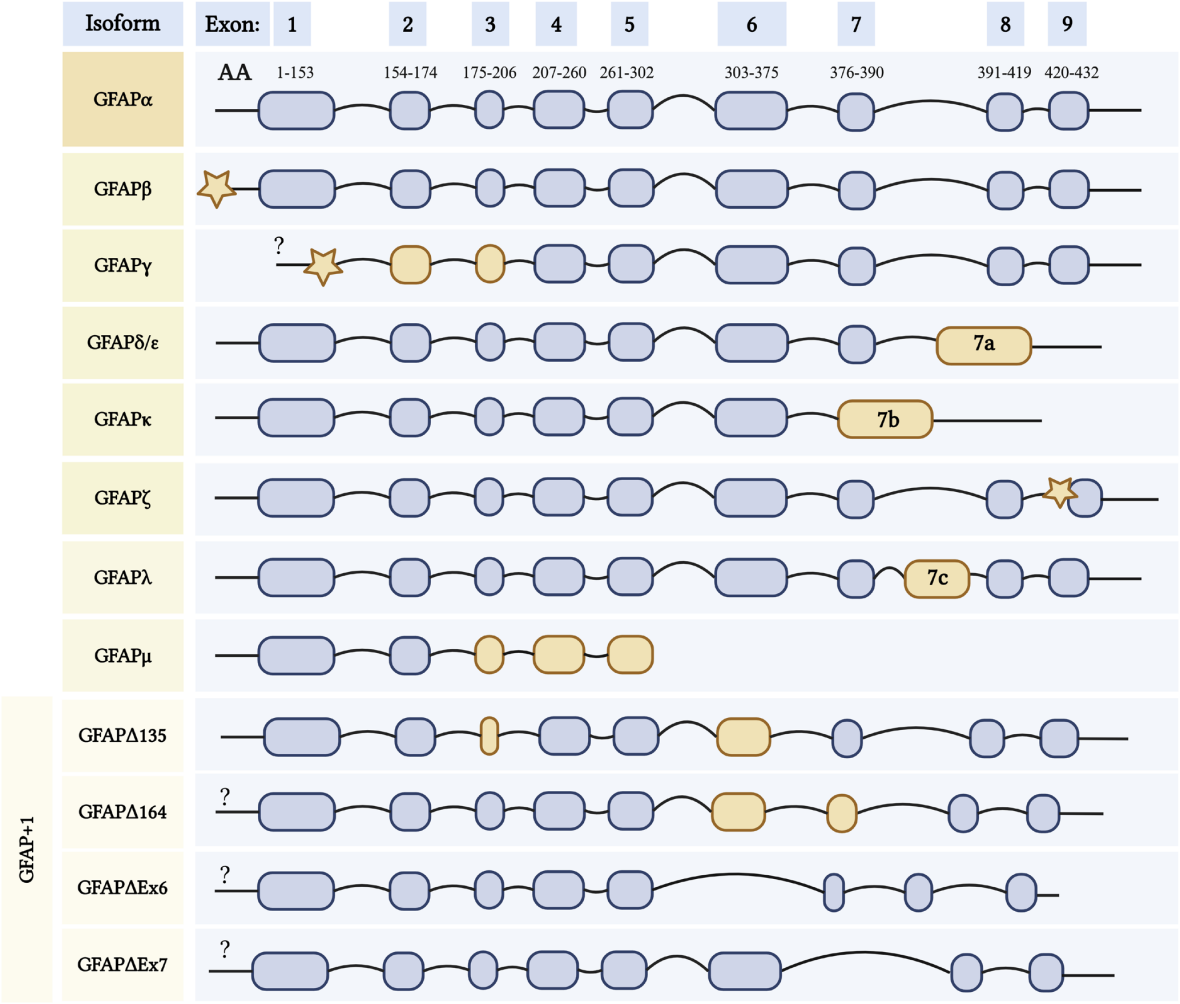
Twelve known isoforms of human GFAP. GFAP*α* (canonical isoform) comprises 9 exons represented as rounded rectangles. Respective exons and splice junctions were mapped to the amino acid sequence of GFAP*α* using the CCDS (NCBI) database (Pujar et al., 2018). GFAP*β* and *γ* isoforms originate from alternative transcription start sites and have a varied N‐terminal (head). The rest of the isoforms result from alternative splicing: *δ* /*ε*, *κ*, and *μ* have shortened C‐terminal (tail), *λ* and *ζ* have alternate rod domains and less common GFAP +1 isoforms have shortened rod domains. Stars represent introns and yellow rounded rectangles indicate alternate regions. Longer linkers in the last two cases of GFAP+1 isoforms indicate that GFAP∆Ex6 lacks exon 6 and GFAP∆Ex7 lacks exon 7. Question marks indicate that the exact start sites of these isoforms are not yet known. Adapted from Middeldorp & Hol (2011), de Reus et al. (2024), and Yang & Wang (2015).

Twelve different human and seven murine GFAP isoforms have been described to this day (de Reus et al., 2024; van Asperen, Robe, & Hol, 2022): *α*, *β*, *γ*, *δ* /*ε*, *κ*, *ζ*, *λ*, *μ,* ∆135, ∆164, ∆exon6, ∆exon7 (de Reus et al., 2024; Kamphuis et al., 2012; van Asperen, Robe, & Hol, 2022; Yang & Wang, 2015; Figure 4). Some of the isoforms have an alternate head (*β* *γ*), tail (*δ* /*ε*, *κ*, *μ*), rod (*ζ*, *λ*), or shortened (GFAP+1) rod domains affecting filament assembly. Notably, for many of the isoforms, full‐length RNAs have not been described and it is not always known where the transcripts start and end (indicated with question marks in Figure 4). The most predominant in the brain and spinal cord and most often studied isoform is 432 AA long GFAP*α* synthesised from 9 exons of the GFAP gene (Middeldorp & Hol, 2011). GFAP*β* (>432 AA) is expressed in Schwann cells and includes a sequence before exon 1 originating in the 5′ untranslated region. The levels of GFAP*β* were shown to be associated with neuronal injury (Condorelli et al., 1999). GFAP*γ* (<432 AA) similarly includes a sequence before exon 1 and has an intron instead of exon 1 (Zelenika et al., 1995). GFAP*δ* /*ε* is the second most common 431 AA long isoform that includes an extra exon 7a and lacks exons 8 and 9 (de Reus et al., 2024).

AA long isoform that includes an extra exon 7a and lacks exons 8 and 9 (de Reus et al., 2024). Increased GFAP*δ* /*ε* expression was detected in human astrocytic tumours reporting a direct correlation between the tumour malignancy and the isoform levels (Choi et al., 2009). GFAP*κ* is the third most commonly investigated isoform, which is 328 AA long and is enriched in the Rosenthal fibres of post‐mortem brains of AxD patients (Lin et al., 2021). GFAP*κ* lacks exons 8 and 9 but contains exon 7b, which consists of exon 7 and intron 7a (de Reus et al., 2024; Yang & Wang, 2015). GFAP*ζ* (>438 AA) includes an intron between exon 8–9 (Kamphuis et al., 2012). AxD mutations result in over‐expression of GFAP*λ* (472 AA), which contains an altered exon 7c (de Reus et al., 2024; Helman et al., 2020). GFAP*μ* (179 AA) has the shortest coding sequence among the known isoforms and is expressed in healthy brain tissue, glioma cell lines, and primary glioma cells (van Bodegraven et al., 2021). Skipping exon 2 results in an out‐of‐frame transcript with a premature termination codon in exon 3 (van Bodegraven et al., 2021). Four less common GFAP isoforms—GFAP+1 collectively refer to variants caused by a single frame‐shift: ∆135 with shortened exon 6 lacking Coil 2B (374 AA), ∆164 with shortened exon 6, 7 and Coil 2B (366 AA), ∆exon6 lacking exon 6 and Coil 2B (347 AA), and ∆exon7 lacking exon 7 (418 AA; Yang & Wang, 2015). The role and abundance of different isoforms in neurodegenerative diseases have not yet been investigated, other than GFAP*α* and GFAP*δ* being elevated in AD brains (Kamphuis et al., 2014). A focused proteomic analysis of GFAP isoforms in neurodegenerative diseases could aid in understanding the role of these different isoforms per disease and potentially pinpoint discovering disease‐specific GFAP isoforms to develop into novel biomarkers.

### 5.2 Post‐translational modifications

PTMs functionally regulate intermediate filament formation (Snider & Omary, 2014) and GFAP is no exception. As shown in Figure 3c, GFAP is heavily modified throughout its sequence, and these PTMs are key in determining the localisation, activity and assembly of the protein. PTMs that lie in epitope regions can potentially hinder the binding of antibodies or the pairs thereof. To this end, the impact of PTMs on the analytical performance and clinical use of different GFAP immunoassays has been poorly characterised (Abdelhak et al., 2022).

Phosphorylation is one of the major PTM types involved in the (dis)assembly of GFAP polymers. Phosphorylation of GFAP in the head domain (Thr‐7, Ser‐8, Ser‐13, Ser‐17 and Ser‐34) regulates the filament disassembly during mitosis (Battaglia et al., 2019). Phosphorylation of Ser‐8 is thought to affect binding to 13‐3‐3*γ* (Li et al., 2006). Increased Ser‐13 phosphorylation is implicated in several pathologies, including disease severity in AxD (Battaglia et al., 2019), disease progression in frontotemporal lobar degeneration (FTLD; Herskowitz et al., 2010), and associated with hypoxic–ischemic brain injury (Sullivan et al., 2012). Furthermore, proteoforms of GFAP which are phosphorylated and N‐glycosylated, are increased in the frontal cortices of AD patients compared to age‐matched controls, whereas isoforms which are O‐glycosylated, showed no such difference (Korolainen et al., 2005). Moreover, GFAP in Rosenthal fibres of AxD patients and rodent models was shown to be ubiquitylated suggesting its critical role in GFAP aggregation (Lin et al., 2024). Another interesting PTM is citrullination, representing an enzymatic deimination forming citrulline from arginine. GFAP is believed to be one of the major deiminated proteins in both health and disease (Brenner & Nicholas, 2017). Citrullination was proposed to influence GFAP filament formation (Inagaki et al., 1989). Peptidylargenine deaminase 2 (PAD2) is an enzyme responsible for the citrullination of GFAP and the amount of PAD2 and citrullinated GFAP is increased in the hippocampi of AD patients compared to non‐AD controls (Ishigami et al., 2015). Additionally, GFAP citrullination was suggested to be a result of an immune response to inflammation in MS (Faigle et al., 2019). As such, citrullinated GFAP is increased in the brains of secondary progressive multiple sclerosis patients compared to controls (Nicholas et al., 2004). Lastly, lipoxidation is another PTM that can potentially affect GFAP polymerisation. The only cysteine at position 294 is susceptible to lipoxidation and is believed to be involved in filament formation (Viedma‐Poyatos et al., 2018). Its mutation to serine has been shown to affect the formation of the cytoskeletal network, suggesting that lipoxidation of this cysteine residue might lead to a similar outcome (Messing & Brenner, 2020; Viedma‐Poyatos et al., 2018). Furthermore, in vitro and cell‐based studies demonstrate that cystine‐generating mutations promote GFAP crosslinking by cysteine‐dependent oxidation, resulting in defective GFAP assembly and decreased filament solubility (Lin et al., 2024). Cys‐291 (mouse GFAP) is palmitoylated in vitro and in vivo and hyper‐palmitoylation was shown to accelerate astrogliosis and neurodegenerative pathology in PPT1‐deficient mice (Yuan et al., 2021). To enhance the reliability of GFAP detection strategies, it is key to consider PTMs during antibody selection. Using a combination of antibodies that target different sites, including those less likely to be affected by known modifications, may improve assay sensitivity and specificity.

### 5.3 Discharge mechanisms

The discharge of GFAP from the brain to the CSF and blood could occur via multiple pathways, which may underlie the established difference in diagnostic performance between plasma and CSF GFAP (Benedet et al., 2021). One study has demonstrated that GFAP efflux into the blood occurs via the glymphatic system in murine models (Plog et al., 2015). Within the glymphatic system, there is first an influx of CSF through AQP4 channels on astrocytes to the interstitial space. This influx of CSF and interstitial fluid in the brain parenchyma then drives a fluid efflux to the perivascular space and venous system (Jessen et al., 2015).

Other hypothesised discharge mechanisms of GFAP, which have yet to be proven, include direct secretion of GFAP from reactive astrocytes to the bloodstream since astrocytic end‐feet surrounds blood capillaries in the brain (Giannoni et al., 2018). Another proposed mechanism is that GFAP may diffuse from the cytosol of injured astrocytes across the blood–brain barrier which is altered and can become ‘leaky’ or damaged in the context of many types of dementia (Hussain et al., 2021), traumatic brain injury (Plog et al., 2015) and stroke (Dvorak et al., 2009). This hypothesis is supported by evidence comparing serum GFAP levels of intracerebral haemorrhage patients, who experience rapid blood–brain barrier disruption, to ischaemic stroke patients, where the opening of the blood–brain barrier occurs more gradually (Dvorak et al., 2009). From 2 to 6 h following stroke onset, serum GFAP was significantly increased in intracerebral haemorrhage patients compared to ischaemic stroke patients. Elevation of GFAP occurred at a much later time‐point of 48 h in ischaemic stroke patients (Dvorak et al., 2009). Another potential mechanism of GFAP release could occur via extracellular vesicles (EVs) since GFAP has been previously detected and quantified in EVs (Flynn et al., 2021). Moreover, GFAP has a high probability of being EV‐associated based on various physio‐chemical properties and PTMs according to a recently developed machine learning model (Waury et al., 2024), suggesting that GFAP is likely to be actively transported through vesicles.

### 5.4 Breakdown products

The full‐length intact 50 kDa GFAP is highly susceptible to proteolysis by calpain and caspase enzymes, by which it is mainly processed to 42 and 38 kDa breakdown products (Escartin et al., 2021; Figure 3d). These generated fragments have different stabilities, ranging from seconds to 20 h, depending on the amino acids that are exposed during cleavage (Phillips et al., 2023). Following calpain enzyme proteolysis, predicted GFAP cleavage sites expose residues, such as serine and alanine, which stabilise the product and cause it to be long‐lived; this suggests these breakdown products may have an important functional role (Phillips et al., 2023).

In a clinical context, two independent studies demonstrated the presence of 36–44 kDa GFAP breakdown products in AD brains (Korolainen et al., 2005; Porchet et al., 2003). GFAP fragments have also been detected in human biofluids, specifically a 38 kDa breakdown product was detected in the CSF (Yang et al., 2022) and plasma (Okonkwo et al., 2013) of patients with TBI within the first 24 h post‐incident. Measurement of total GFAP and its breakdown products in the serum of TBI patients aided in the diagnosis of intracranial injury compared to clinical screening alone (McMahon et al., 2015). A comparison of the 38 kDa GFAP proteolytic fragment versus intact GFAP measured in CSF to distinguish between TBI patients versus controls showed better discriminative performance for the 38 kDa fragment compared to full‐length GFAP (AUC‐ROC of 0.944 versus 0.909; Yang et al., 2022). Interestingly, phosphorylation of GFAP in AxD leads to proteolytic cleavage by caspase 6, resulting in fragments of varying molecular weight compared to the 38 kDa product, which is only produced following acute injury (Battaglia et al., 2019). Different GFAP breakdown products may therefore define acute astrocyte injury, in the context of TBI, versus chronic injury in the context of neurodegenerative conditions.

### 5.5 Aggregation, dynamics and sample stability

Several GFAP‐isoforms have a high propensity to form Rosenthal fibre‐like aggregates and a high percentage of such isoforms can lead to an IF‐network collapse (de Reus et al., 2024; Lin et al., 2021). In AxD, a specific mutation in GFAP alters splicing, leading to an increase in aggregation‐prone isoforms: GFAP‐*δ*, −*κ* and ‐*λ* (Lin et al., 2021). These isoforms are less soluble compared to non‐pathological GFAP, making them prone to form rod‐shaped proteinaceous aggregates inside astrocytes called Rosenthal fibres. The fibres interfere with cell mitosis, alter the morphology of aggregate‐bearing astrocytes and are the pathological hallmark of AxD (Lin et al., 2021). They are also sometimes present in MS (Wippold et al., 2006) and glioma (Gullotta et al., 1985). Rosenthal fibre‐like aggregates can be extraordinarily stable and this may limit the detection of GFAP in blood in AxD (Abdelhak et al., 2022). Aggregation or de‐aggregation of GFAP may occur in different matrices and/or in response to temperature changes; GFAP levels in blood were shown to increase with storage or freeze–thaw (FT) cycles at −20°C (Gouda et. al, in prep) or at −80°C (Verberk et al., 2022). However, there is no direct evidence that this increase in the level of GFAP monomers occurs because of protein de‐aggregation. Conversely, CSF GFAP levels have been shown to decrease with FT cycles, and CSF GFAP was shown to be more susceptible to FT cycles compared to blood GFAP measured in the same individual (Simrén et al., 2022). However, even in fresh samples, GFAP measured in blood was superior to CSF measurements in discriminating between amyloid‐positive and amyloid‐negative individuals (thereby reflecting AD pathology), suggesting that the matrix discrepancy is likely not solely because of sample stability and alterations in GFAP aggregation. A recent study has highlighted structural heterogeneity and the existence of multiple conformational forms of GFAP (Gogishvili et al., 2023). The study explored the structural dynamics of recombinant GFAP under three conditions, namely potassium phosphate buffer saline (KPBS) and two setups in artificial CSF (aCSF) using hydrogen‐deuterium exchange mass spectrometry. Under aCSF conditions, recombinant GFAP showed an overall increase in solvent accessibility and simultaneously displayed hotspots of aggregation, suggesting the existence of multiple conformations of GFAP (Gogishvili et al., 2023).

### 5.6 Interaction partners

Protein‐antibody accessibility can also be hampered because of interacting partners. Intermediate filament‐associated proteins play an important role in filament stability and facilitate links to other structures within the cell (Middeldorp & Hol, 2011). Plectin is a widely expressed IF‐binding protein, which is thought to provide mechanical strength to cells by cross‐linking to microtubules and the actin cytoskeleton, and was shown to bind to GFAP in the rod domain (Tian et al., 2006). Additionally, decreased plectin levels lead to the formation of a disorganised aggregate of GFAP (severe type of AxD mutation, R239C (RC); Tian et al., 2006). GFAP was shown to interact with the family of regulatory proteins 14‐3‐3 and that the interaction is influenced by the phosphorylation of GFAP in a cell‐cycle dependent manner (Li et al., 2006). Small Heat shock proteins (sHSPs) are a group of ATP‐independent chaperones expressed ubiquitously in all kingdoms of life (Haslbeck et al., 2019) and play a key role in preventing protein misfolding and aggregation (Haslbeck & Vierling, 2015). Upon stress, HSP27 has a phosphorylation‐activated role in actin filament regulation and prevents filament disruption and degeneration (Graceffa, 2011; Guay et al., 1997). Moreover, sHSPs are involved in cytoskeletal rearrangement. HSP27 along with *α*B‐crystallin was shown to interact with GFAP regulating filament assembly (Perng et al., 1999). Increasing GFAP*δ* levels by transient transfection in astrocyte‐derived cell lines were shown to have deleterious effects, causing the increased association of *α*B‐crystallin and the disruption of IF network (Perng et al., 2008). Moreover, HSP27 was found in Rosenthal fibres (GFAP inclusions) both in the brains of patients suffering from AxD (Tomokane et al., 1991), as well as in mice overexpressing GFAP (Eng et al., 1998).

## 6 DISCUSSION

The comprehensive evaluation of GFAP has the potential to enable longitudinal evaluation of the astrocyte response in brain and spinal cord disorders. A better understanding of GFAP proteoforms can ultimately assist with the development of accurate, early and discriminative diagnosis. There is much to discover about the implications of GFAP proteoforms and accessibility in the context of neurodegeneration. To better understand disease pathology, improve the utilisation of GFAP as a biomarker and unlock various biological insights, we need to have a comprehensive understanding of what we are detecting and quantifying in biomarker tests.

GFAP (post‐)transcriptional regulation has a key role in glial cell physiology and pathology, as GFAP isoforms vary in cellular localisation and determine mechanical properties of the IF‐network (de Reus et al., 2024). There are numerous open questions regarding the implications of distinct GFAP isoforms, concerning the function of GFAP isoforms in ageing, brain injury and disease. In more detail, using isoform‐specific antibodies may hold promise for staging AD in terms of inflammation; the shortened rod isoforms (GFAP+1) are associated with disease progression as GFAP+1 positive astrocytes have been shown to increase in number over the course of AD (Kamphuis et al., 2014). Another interesting avenue to investigate is the GFAP isoform ratio. Amyloid precursor protein (APP)‐derived peptides exemplify the case where the ratio of A*β* 42 to A*β* 40 (A*β* 42/40 ratio) is superior to the concentration of A*β* 42 alone in discriminating patients with AD from controls (Shoji et al., 1998). A lower ratio is indicative of disrupted amyloid metabolism and is used as a diagnostic tool for AD (Dumurgier et al., 2015; Perez‐Grijalba et al., 2019). For GFAP isoform ratios, a change in the GFAP*α*/GFAP*δ* ratio has been shown to alter cell‐environment interactions and cell migration in the context of glioma cell invasion (van Asperen, Robe, & Hol, 2022). Importantly, glioma does not directly translate to dementia and there are major differences in pathological processes. Yet, it may be valuable to investigate the role of the GFAP*α*/GFAP*δ* ratio to reflect different states of astrocyte activation in the context of dementias. The interplay between these isoforms and their differential effects on protein aggregation and neurotoxicity highlights the importance of understanding the GFAP proteoform landscape for unravelling the complexity of neurodegenerative disorders and advancing biomarker research.

PTMs play a pivotal role in regulating the functional properties of GFAP (Snider & Omary, 2014). Among others, phosphorylation regulates the assembly and disassembly of GFAP polymers, binding to other proteins and disease severity (Battaglia et al., 2019; Herskowitz et al., 2010; Li et al., 2006; Sullivan et al., 2012). PTMs affecting filament formation can impact the accessibility of GFAP to antibodies used in assays, through obscuring or exposing epitopes leading to variability in assay results. Understanding the complex cross‐talk and regulatory mechanisms of these PTMs is crucial for unravelling their functional significance and developing PTM‐specific biomarker tests for differential diagnosis and disease staging (Battaglia et al., 2019; Herskowitz et al., 2010). The importance of detecting specific PTMs for differential dementia diagnosis can be highlighted with the example of phosphorylated tau. Phosphorylated tau has long been established to reflect abnormal tau metabolism in the brain. The identification and quantification of specific phospho‐tau epitopes have proven instrumental in elucidating disease diagnosis, progression and severity. For instance, CSF p‐tau181 is one of the core biomarkers for AD diagnosis (Olsson et al., 2016). Recently, CSF p‐tau217 has been shown to perform better for diagnostic workup in AD (Janelidze et al., 2020). Both plasma p‐tau231 and p‐tau217 were shown to associate with the earliest cerebral A*β* pathologies (Milà‐Alomà et al., 2022), implicating their role in early diagnosis. The potential of phospho‐GFAP or other PTMs as biomarkers for differential dementia diagnosis has not yet been explored, but based on research summarised above, phospho‐GFAP Serine 13 could be a potential FTLD‐specific biomarker test, and citrullinated GFAP may hold promise for AD and/or MS.

GFAP clearance mechanisms and transport from the brain to the CSF and blood could underly differences in diagnostic performance between plasma and CSF GFAP. The contribution of each of the hypothesised mechanisms and their disruption could impact the performance of GFAP assays in different biological matrices. For example, traumatic brain injury reduces clearance via the glymphatic system which has been shown to suppress TBI‐induced increases of GFAP in the blood, which negatively impacts its clinical utility (Plog et al., 2015). Contrarily, a disrupted blood–brain barrier in AD could enhance the discharge of GFAP to the blood, underlying its superior performance compared to CSF. Further work studying GFAP dynamics in both CSF and blood matrices, in combination with MRI scans of blood–brain barrier quality in the context of various diseases with different dynamics of astrocyte injury can help elucidate the contribution of different proposed pathways.

Given the susceptibility of full‐length GFAP to proteolysis, it is crucial to understand its cleavage products. It is yet to be determined exactly how GFAP fragments are released from astrocytes for detection in CSF and blood, whether they are related to the pathogenesis and progression of the disease, and whether they outperform full‐length GFAP as stand‐alone biomarkers. Breakdown products have different stabilities compared to the native full‐length protein depending on the residues exposed on the breakdown products (Phillips et al., 2023), and potentially the matrix they are in. Investigating the differences in the abundance of specific GFAP breakdown products among biological matrices in various disease contexts could elucidate this. Additionally, breakdown products may have distinct functions compared to native proteins and could act to serve as stand‐alone biomarkers, as is the case for TBI (Yang et al., 2022), and they may reflect different cellular processes and disease pathologies. The development of novel breakdown product‐specific immunoassays would help answer these questions. In the case of NfL, which is another intermediate filament protein that is widely used as a biomarker for axonal damage, characterisation of the Uman antibodies used in commercially available immunoassays revealed their neurodegeneration‐specific staining properties (Shaw et al., 2023). Surprisingly, neither antibody stains neurofilaments in healthy cells but rather recognises degenerated neuronal NfL. This study highlights the importance of targeting protein products which are specifically produced in the context of neurodegeneration rather than constitutively produced.

GFAP is a highly dynamic and flexible protein co‐existing in multiple conformational species. Focusing on specific oligomeric or aggregated GFAP species can provide valuable insights into the severity of the disease. Under specific conditions, GFAP may become more disordered and *floppy* (as shown in the case of artificial CSF; Gogishvili et al., 2023), which increases solvent accessibility, especially in the interface regions, possibly leading to amorphous aggregation. The structural dynamics of GFAP can affect the performance of immunoassays potentially in the context of both different matrices or buffers used in the assay protocol.

In future studies, cross‐linking mass spectrometry and hydrogen‐deuterium exchange mass spectrometry techniques can be explored for mapping the binding interface of GFAP and to better understand GFAP solvent accessibility. This can help elucidate optimal epitope sites for antibody binding when generating novel immunoassays. Furthermore, understanding which proteins GFAP binds to in the context of different diseases can help us gain insight as to the exact biological process the biomarker is reflecting.

## 7 CONCLUSION

GFAP has been proven to be a highly valuable addition to the biomarker toolbox for early and discriminative diagnosis of brain and spinal cord disorders. By taking an example of other successful biomarkers developed for various neurological diseases, we can harness the GFAP proteoform diversity to develop more accurate and disease‐specific biomarkers (see Concluding remarks). Proteoform‐specific targeting (including isoforms, PTMs, discharge mechanisms, breakdown products, and higher‐order species) could reveal novel biological insights, which might lead to more reliable and reproducible tests and improved diagnostic performance.

### 7.1 Concluding remarks

Potential avenues to explore for advancing GFAP as a biomarker:

- *Isoforms* can provide valuable information about the underlying CNS pathology. For instance, GFAP+1 isoforms may hold promise for staging AD in terms of inflammation, while GFAP*α*/GFAP*δ* ratio may reflect different states of astrocyte activation.
- *Post‐translational modifications (PTMs)* of GFAP can be exploited by developing PTM‐specific biomarker tests that could aid in differential diagnosis and/or disease staging. For example, phosphorylated GFAP may be useful for identifying AxD, AD, or FTLD, while citrullinated GFAP could help diagnose AD and MS.
- *Discharge mechanisms* of GFAP from the brain to the CSF and blood could underlie differences in diagnostic performance between plasma and CSF GFAP. Additionally, clearance mechanisms have clinical relevance for utilising GFAP as a biomarker of acute astrocytic injury, such as for TBI.
- *Breakdown products* of GFAP may exhibit distinct functions compared to full‐length GFAP. Furthermore, different cleavage products could serve as markers for acute versus chronic astroglial injury.
- *Aggregates or higher‐order species* of GFAP are associated with disease progression and may serve as useful biomarkers for AxD and potentially MS patients. Moreover, detecting specific oligomeric or aggregated GFAP species could provide valuable insights into the severity of the disease.
- *Interaction partners* of GFAP can influence protein‐antibody accessibility, potentially affecting biomarker test performance in specific matrices. Understanding the GFAP interactome in different pathological conditions can provide crucial biological insights into the mechanisms underlying disease progression.

## AUTHOR CONTRIBUTIONS

**Dea Gogishvili:** Conceptualization; investigation; visualization; writing – original draft; writing – review and editing. **Madison I. J. Honey:** Conceptualization; investigation; writing – original draft; writing – review and editing. **Inge M. W. Verberk:** Conceptualization; writing – original draft; writing – review and editing. **Lisa Vermunt:** Conceptualization; writing – original draft; writing – review and editing. **Elly M. Hol:** Writing – original draft; writing – review and editing. **Charlotte E. Teunissen:** Conceptualization; funding acquisition; writing – original draft; writing – review and editing. **Sanne Abeln:** Conceptualization; funding acquisition; writing – original draft; writing – review and editing.

## CONFLICT OF INTEREST STATEMENT

CT is the recipient of ABOARD, which is a public‐private partnership receiving funding from ZonMW (#73305095007) and HealthHolland, Topsector Life Sciences & Health (PPP‐allowance; #LSHM20106). More than 30 partners participate in ABOARD. ABOARD also receives funding from Edwin Bouw Fonds and Gieskes‐Strijbisfonds. CT has a collaboration contract with ADx Neurosciences, Quanterix and Eli Lilly, performed contract research or received grants from AC‐Immune, Axon Neurosciences, Biogen, Brainstorm Therapeutics, Celgene, EIP Pharma, Eisai, Olink, PeopleBio, Roche, Toyama, Vivoryon. CT serves on editorial boards of Medidact Neurologie/Springer, Alzheimer Research and Therapy, Neurology: Neuroimmunology & Neuroinflammation, and is editor of a Neuromethods book Springer. LV is supported by research grants from Amsterdam UMC, ZonMw, Stichting Diorapthe, and Olink and consultancy/speaking fees from Roche and Eli Lilly, all paid to her institution. Outside the submitted work: SA reports a patent pending; SA is in a consortium agreement with Cergentis BV as part of the TargetSV project; SA is in a consortium agreement with Olink and Quanterix as part of the NORMAL project. The rest of the authors do not have any competing interests to declare.

### PEER REVIEW

The peer review history for this article is available at https://www.webofscience.com/api/gateway/wos/peer‐review/10.1111/jnc.16226.

## Data Availability

The data that support this study are openly available. Several tools were employed to analyse the structure of GFAP, namely, NetSurfP 3.0 (Høie et al., 2022; Klausen et al., 2019), AlphaFold‐Multimer (Evans et al., 2021; Gogishvili et al., 2023), and the PAE viewer tool (Elfmann & Stülke, 2023). The Human Protein Atlas (HPA) was utilised for the analysis of GFAP expression, interaction network, and cell localisation (Karlsson et al., 2021; Kawajiri et al., 2003; Thul et al., 2017; Uhlén et al., 2015; Yoshida et al., 2007). The feature overview of GFAP was obtained from curated UniProt annotations (Consortium, T. U, 2023). For mapping exons and splice junctions to the amino acid sequence of GFAP*β*, the NCBI‐CCDS database was used (Pujar et al., 2018). Figures 3d and 4 were created with Biorender.com.
